# Development of a Stage- and Species-Specific RNAi System for Molecular Insights in *Trichogramma* Wasps

**DOI:** 10.3390/insects16070673

**Published:** 2025-06-27

**Authors:** Zelong Yang, Yan Lu, Zhuo Jiang, Xilin Jiao, Han Lin, Wanning Jiang, Wenmei Du, Xue Zhang, Zhao Peng, Junjie Zhang, Xiao Wang, Ying Hu

**Affiliations:** 1 Engineering Research Center of Natural Enemies, Institute of Biological Control, Jilin Agricultural University, Changchun 130118, China; 17731008475@163.com (Z.Y.); luyan20020624@163.com (Y.L.); jzjz8968@163.com (Z.J.); j18943100339@163.com (X.J.); 15568628611@163.com (H.L.); winniejiang1008@163.com (W.J.); wenmeid@jlau.edu.cn (W.D.); zhangxue871013@163.com (X.Z.); zhangjunjie9777@jlau.edu.cn (J.Z.); 2 Department of Plant Pathology, College of Plant Protection, Jilin Agricultural University, Changchun 130118, China; zpeng21@jlau.edu.cn

**Keywords:** *Trichogramma* wasps, RNA interference, soaking, microinjection, species-specific optimization

## Abstract

*Trichogramma* wasps, egg parasitoids widely used to control lepidopteran pests, have long eluded in-depth molecular mechanistic studies due to their minute size and genetic tool scarcity. While previous RNAi efforts were restricted to *T. dendrolimi*, we developed the first cross-species RNAi system for both *T. dendrolimi* and the previously intractable *T. ostriniae*. Temporal expression profiling identified *white* and *laccase 2* as stage-specific RNAi targets, peaking during prepupal/pupal stages, which were tested across species and developmental stages using microinjection and soaking dsRNA delivery methods. Survival analysis prioritized soaking for *T. dendrolimi* prepupae/pupae, while microinjection was essential for *T. ostriniae* to bypass prepupal mortality during soaking. Concentration-dependent RNAi targeting the *white* gene achieved 85.61% transcript reduction in *T. dendrolimi* via soaking and 89.36% in *T. ostriniae* via microinjection at 2000 ng/μL, correlating with 64.06% and 32.09% white-eyed pupae, causing a significant reduction in eye pigments. For the *laccase 2* gene, soaking at 2000 ng/μL induced 88.35% transcript reduction in *T. dendrolimi* and 73.31% in *T. ostriniae*, leading to incomplete cuticle tanning and sclerotization. This study resolves the long-standing challenge of genetic manipulation in *Trichogramma* wasps, providing a universally applicable framework to decipher parasitoid–host interactions at the molecular scale, which is useful for sustainable pest management strategies.

## Simple Summary

*Trichogramma* wasps, as environmentally friendly biocontrol agents, are widely employed to control lepidopteran pests. In recent years, people have attempted to study their gene function through molecular means in order to improve the control efficiency against agricultural and forestry pests of *Trichogramma* wasps. However, the progress has been severely hindered by their minute body size (<1 mm) and complex host egg-dependent parasitic behavior. In this study, we established a universal RNA interference (RNAi) technology system applicable to *T. dendrolimi* and *T. ostriniae*, targeting the *white* gene (regulating eye pigment deposition) and *laccase 2* gene (involved in cuticle sclerotization). Furthermore, we analyzed the effects of different dsRNA treatment concentrations and treatment periods on interference efficiency, overcoming the technical barriers associated with cross-species applications in conventional methods. Phenotypic analyses revealed that both non-invasive soaking and microinjection approaches achieved efficient gene silencing in *T. dendrolimi* and pupal-stage *T. ostriniae*. Notably, to avoid high mortality rates, pre-pupal *T. ostriniae* is required to use the microinjection method. This work establishes standardized protocols for functional gene studies in miniature parasitoid wasps and provides methodological foundations for developing precision biocontrol technologies through genetic engineering.

## 1. Introduction

*Trichogramma* wasps, serving as critical egg parasitoids in global lepidopteran pest management, have achieved remarkable ecological success through decades of field implementation [1,2]. In Northeast China, the release of *T. dendrolimi* protects over 4 million hectares of cornfields annually against the Asian corn borer (*Ostrinia furnacalis*), covering 35% of regional maize cultivation [2]. While extensive applied research has focused on ecological optimization and breeding technology enhancements, a fundamental disconnect persists between empirical biocontrol practices and mechanistic understanding due to a critical knowledge gap perpetuated by the lack of robust genetic tools in this parasitoid wasp.

RNA interference (RNAi) technology has become an important tool for genetic function research in various insects [3]. However, its efficiency is constrained by delivery methods [4], commonly dsRNA delivery methods including feeding, microinjection, and soaking; each presents distinct advantages and limitations shaped by developmental biology constraints. New delivery methods are being developed, e.g., through nanocarriers [5,6]. Feeding-based RNAi, while offering simplicity and minimal invasiveness for insects, is inherently restricted to feeding-active stages and exhibits delayed efficacy, rendering it ineffective during non-feeding phases such as embryogenesis or pupal development [7]. Microinjection, the most widely utilized method, enables precise dsRNA delivery across all ontogenetic stages from embryos to adults, achieving high phenotypic penetrance [8]. However, this technique demands specialized expertise and induces significant mechanical stress, with high mortality rates due to injection trauma [9]. Soaking protocols, involving the immersion of permeable developmental stages (e.g., larvae/pupae) in dsRNA solutions, provide a technically accessible alternative but require higher dsRNA concentrations than microinjection to attain comparable silencing efficiency [10]. This methodological triad underscores a critical trade-off in parasitoid RNAi research: balancing operational feasibility against biological precision and organismal viability.

The application of RNAi in insects exhibits profound taxonomic divergence, with Coleopteran insects demonstrating robust systemic silencing compared to Lepidoptera and Diptera [11]. Within Hymenopteran parasitoids, RNAi advancements have been predominantly confined to larger-bodied larval and pupal parasitoids. For instance, silencing the *doublesex* gene in early male larvae of *Nasonia vitripennis* using microinjection impacts the growth and differentiation of reproductive tissue [12]. In the fruit fly parasitoid *Asobara japonica*, the microinjection of dsRNA targeting the *ebony* gene successfully alters the body color [13]. However, these methods face significant technical challenges in minute egg parasitoids like *Trichogramma*, which are typically smaller than 1 mm. Most of the current RNAi research in *Trichogramma* has focused on *T. dendrolimi*. Microinjection combined with nanocarriers enables *VgR* silencing in prepupae, disrupting ovarian development [14], while pupal-stage injections achieve *Ferhch* knockdown, causing wing deformities [15]. Moreover, RNAi targeting *chitinase 10*, *v-ATPase A*, and *v-ATPase B* genes has been achieved through microinjection at the pupa stage, while feeding larvae and adults with dsRNA failed to silence these genes, underscoring the ineffectiveness of feeding as a delivery method for RNAi in *T. dendrolimi* [16]. Despite these advances, the application of RNAi in these small parasitoids is still hindered by several limitations. Microinjection, though effective, causes mechanical damage to the tiny individuals, resulting in high mortality rates [17]. More importantly, existing technologies are not adaptable to *T. ostriniae* (body size <0.5 mm) reared on eggs of rice meal moth *Corcyra cephalonica* or Asian corn borer *O. furnacalis*, as their size differences and host specificity make traditional approaches difficult to apply across species.

This study focuses on *T. dendrolimi* and *T. ostriniae* as dual models, targeting two phenotypically clear, stage-specific genes: *white* (responsible for eye pigment deposition) and *laccase 2* (responsible for epidermal tanning). By comparing the delivery efficiency of microinjection and soaking at different developmental stages (prepupal/pupal) and between species, a standardized operational framework for cross-host and cross-species RNAi delivery is established for the first time. The innovation of this research lies in the construction of the first RNAi technology system applicable to multiple *Trichogramma* species, providing a key tool for analyzing the molecular mechanisms of host adaptability in *Trichogramma*. This technological framework not only advances genetic function research in *Trichogramma* but also offers new ideas and methods for further studying the molecular interactions between *Trichogramma* and its hosts.

## 2. Materials and Methods

### 2.1. Insect Rearing

*T. dendrolimi* and *T. ostriniae* were originally collected from the parasitized eggs of *O. furnacalis* in the corn fields in Yitong, Jilin Province, China (125°11′ E, 43°3′ N) in 2015 and were identified through the morphological characteristics of the male genital capsule and rDNA-ITS2 sequence analysis. *T. dendrolimi* and *T. ostriniae* were reared and maintained on the eggs of *C. cephalonica* under laboratory conditions at 25 ± 1 °C, 75 ± 5% relative humidity, and a photoperiod of 16L:8D. After being continuously reared for several generations on *C. cephalonica* eggs, *T. dendrolimi* and *T. ostriniae* colonies were reared on *Antheraea pernyi* eggs and *O. furnacalis* eggs, respectively, for subsequent RNAi experiments.

### 2.2. Identification and Analysis of White and Laccase 2 Genes

To identify *white* and *laccase 2* genes in *T. dendrolimi* and *T. ostriniae*, BLASTP v2.16.0 search (e-value < 1 × 10^−5^, bit score > 100, identity > 70% and query coverage > 70%) was performed against the genomes of *T. dendrolimi* and *T. ostriniae*, using query sequences from *Drosophila melanogaster* (*white*: NP476787.1; *laccase 2*: NP724412.1), *Bombyx mori* (*white*: NP001037034.1; *laccase 2*: NP001103395.1), and *Apis mellifera* (*white*: NP001403446.1; *laccase 2*: XP006562317.1) retrieved from the NCBI database (http://www.ncbi.nlm.nih.gov/, accessed on 1 April 2023). Multiple sequence alignment of the *white* or *laccase 2* gene sequence of *T. dendrolimi* and *T. ostriniae* with the sequence of *D. melanogaster*, *B. mori*, and *A. mellifera* was performed to check the sequence similarity (Figures S1 and S2).

### 2.3. Temporal Expression Analysis of White and Laccase 2 Genes

To evaluate the temporal expression of *white* (accession number: PV568319.1) and *laccase 2* (accession number: PV568320.1) genes, samples from different developmental stages were collected for total RNA extraction. For *T. dendrolimi*, the fresh *A. pernyi* eggs were inoculated with newly emerged *T. dendrolimi* (<24 h old) for 24 h at a ratio of wasps to host eggs of 1:10. The samples were collected at different developmental stages, including egg (1st day post-parasitism), larva (3rd day post-parasitism), prepupa (5th-6th day post-parasitism), pupa (7th–11th day post-parasitism), and newly emerged adult wasps. For *T. ostriniae*, fresh corn borer egg masses were parasitized with newly emerged *T. ostriniae* (<24 h old) for 24 h at a ratio of wasps to host eggs of 1:6, and samples were collected at different developmental stages, including egg (4 h after parasitism), larva (2nd day post-parasitism), prepupa (4th–5th day post-parasitism), pupa (6th–9th day post-parasitism), and newly emerged adult wasps. For each biological replicate, approximately 1 g of host egg samples containing different developmental stages of *T. dendrolimi* or *T. ostriniae* was collected. In total, three biological replicates were performed for each developmental stage. The samples were rapidly frozen in liquid nitrogen and stored at −80 °C.

Primers for RT-qPCR were designed using the IDT PrimerQuest™ Tool (https://eu.idtdna.com/pages/tools/primerquest) (accessed on 17 October 2024) [18], based on the sequences of the *white*, *laccase 2*, and *GAPDH* genes from both species. The primers are listed in Table 1 and were designed to avoid overlap with synthesized dsRNA regions. Total RNA was extracted using the EASYspin plus Cell/Tissue Total RNA Isolation Kit (Aidlab Biotechnologies Co., Ltd., Beijing, China), and cDNA synthesis was performed using the TRUEscript RT MasterMix (OneStep gDNA Removal) (Aidlab Biotechnologies Co., Ltd., Beijing, China). RT-qPCR was conducted on a qTOWER^3^G system (Analytik Jena, Jena, TH, Germany) using 2× SGExcel FastSYBR Mixture (Sangon, Shanghai, China) under the following conditions: 95 °C for 5 min, followed by 40 cycles of 95 °C for 5 s and 60 °C for 20 s. Relative expression levels were calculated using the 2^−ΔΔCt^ method, with *GAPDH* as the reference gene.

### 2.4. Preparation of dsRNAs

Primers for RNAi fragments targeting the *white* and *laccase 2* genes, with product sizes ranging from 600 to 800 bp, were designed using SnapDragon-sRNA DesignTool (https://www.flyrnai.org/cgi-bin/RNAi_find_primers.pl) (accessed on 10 October 2024.), based on regions of high sequence similarity between these genes in *T. dendrolimi* and *T. ostriniae* (Figures S9 and S10). The primers are listed in Table 2. The T7 promoter sequence (5′-TAATACGACTCACTATAGGG-3′) was added to the 5′ end of all primers. PCR amplification of the dsRNA synthesis templates was performed using Taq PCR Master Mix (Sangon, Shanghai, China), and the products were purified using the Wizard^®^ SV Gel and PCR Clean-Up System (Promega, Madison, WI, USA) and synthesized using the T7 RNA Transcription Kit (ZSYJ, Shanghai, China).

### 2.5. RNA Interference Assay via Soaking and Microinjection Delivery Method

To systematically evaluate RNA interference efficacy across developmental stages and species, we implemented complementary soaking and microinjection protocols for *T. dendrolimi* (reared in *A. pernyi* eggs) and *T. ostriniae* (reared in *O. furnacalis* eggs). For the soaking method, synchronized prepupae and pupae were dissected under stereomicroscopy (Figures S3 and S4) at species-specific post-parasitism timepoints (*T. dendrolimi*: prepupae at 6 days, pupae at 7 days; *T. ostriniae*: prepupae at 4 days, pupae at 6 days) and transferred to Parafilm-based artificial hosts with hemispherical chambers (Figure S5) [19]. Initial survival assays involved 48 h of soaking with 1 μL ddH_2_O or 500 ng/μL ds*GFP*, followed by excess solution removal via filter paper. For each biological replicate, 30 individuals were placed within a single artificial host egg (3 eggs per replicate, each containing 30 wasps), with three independent replicates performed per treatment group (total *n* = 270 per stage/species). Adult emergence was monitored to quantify survival rates. Subsequent silencing optimization comprised two experimental designs: (1) time-course analysis—prepupae of *T. dendrolimi* (6th day post-parasitism) and *T. ostriniae* (4th day post-parasitism) soaked in the ds*white* solution (500 ng/μL and 2000 ng/μL) and pupae of *T. dendrolimi* (7th day post-parasitism) and *T. ostriniae* (6th day post-parasitism) in the ds*laccase 2* solution (500 ng/μL and 2000 ng/μL) for 12, 24, and 48 h; and (2) dose–response profiling—prepupae of *T. dendrolimi* (6th day post-parasitism) and *T. ostriniae* (4th day post-parasitism) exposed to the ds*white* solution (100, 300, 500, 1000, 1500, and 2000 ng/μL) and pupae of *T. dendrolimi* (7th day post-parasitism) and *T. ostriniae* (6th day post-parasitism) to the ds*laccase 2* solution (100, 300, 500, 1000, 1500, and 2000 ng/μL) for 48 h, with ds*GFP* (500 ng/μL) controls. After soaking for 48 h, samples were pooled (30 individuals/replicate, 3 replicates) to check the gene silencing efficiency via RT-qPCR validation.

Parallel microinjection experiments utilized the same developmental stages as soaking treatments to ensure direct methodological comparability: *T. dendrolimi* prepupae (6th day post-parasitism) and pupae (7th day) and *T. ostriniae* prepupae (4th day) and pupae (6th day). Synchronized individuals were immobilized on 2.5% agarose plates, and a PV850 microinjector (WPI) delivered 1 μL ddH_2_O or 500 ng/μL ds*GFP* into the thoraco-abdominal junction at 10.0 PSI to assess baseline survival (total n = 270 per stage/species) (Figure S6). For gene silencing, prepupae were injected with the ds*white* solution (100, 300, 500, 1000, 1500, and 2000 ng/μL) and pupae were injected with the ds*laccase 2* solution (100, 300, 500, 1000, 1500, and 2000 ng/μL), with identical ds*GFP* controls. After 48 h of the treatment, samples were collected (30 individuals/replicate, 3 replicates), and the silencing efficiency was assessed via RT-qPCR. Developmental stage alignment and concentration gradients mirrored soaking protocols to enable direct methodological comparison.

### 2.6. Phenotypic Evaluation

Following 48 h post-treatment intervals (soaking or microinjection with ds*white* solution), eye pigmentation phenotypes were assessed using a VHX-7000 digital microscope (Keyence, Osaka, Japan) at 200× magnification. To standardize the quantification of *white* gene silencing efficacy, a four-tier chromatic classification system based on ommochrome deposition levels was established: Class I (fully pigmented, red), Class II (partial pigmentation, semi-red), Class III (low pigmentation, light red), and Class IV (unpigmented, white). All evaluations were performed on immobilized individuals under consistent illumination parameters (LED ring light, 5600K color temperature) to minimize optical artifacts.

Following 48 h post-treatment intervals (soaking or microinjection with ds*laccase 2* solution), deformations to the cuticle and body segments, as well as a lack of pigmentation, were recorded using a Keyence VHX-7000 digital microscope at 200× magnification. *Trichogramma* wasps were scored as having a knockdown phenotype if we observed one or more of the traits described above.

### 2.7. Statistical Analysis

Statistical analyses were performed using GraphPad Prism 6.0 (GraphPad Software, San Diego, CA, USA). Two-tailed Student’s *t*-test was used to analyze differences between two samples, and one-way ANOVA was performed for multiple group comparisons.

## 3. Results

### 3.1. Temporal Expression Patterns of Two Genes in Two Trichogramma Species

**Figure 1:**
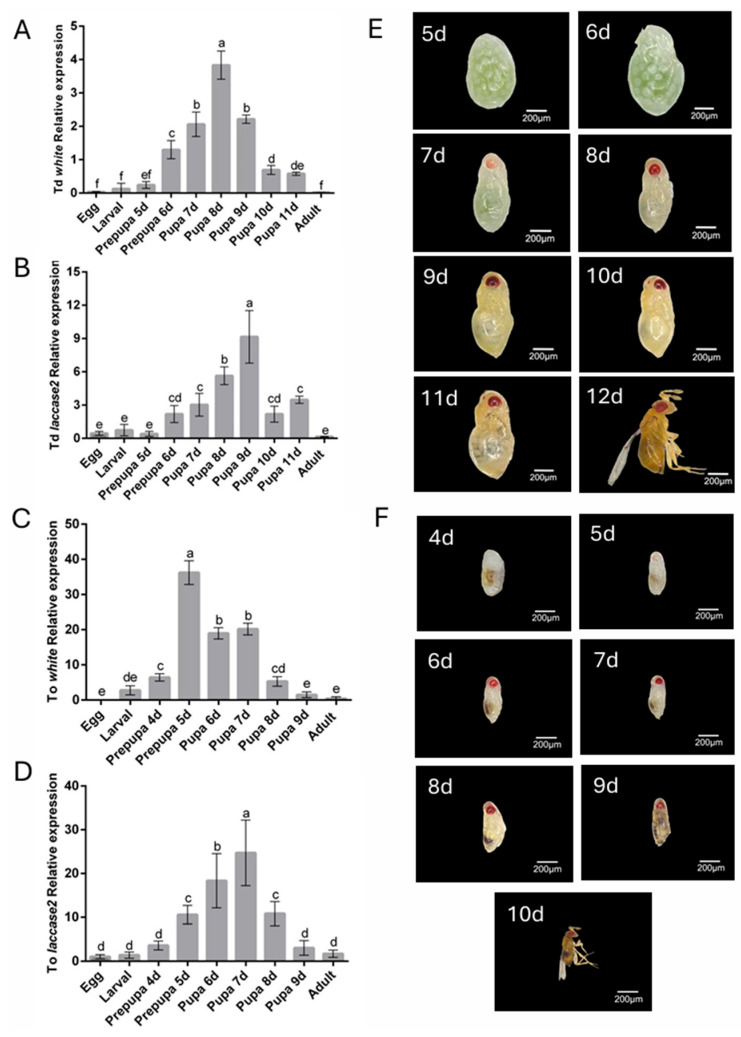
Spatial expression patterns and developmental timelines of *white* and *laccase 2* genes in *T. dendrolimi* (Td) and *T. ostriniae* (To). (**A**) Developmental expression profile of the *white* gene in *T. dendrolimi* analyzed by RT-qPCR. (**B**) Developmental expression profile of the *laccase 2* gene in *T. dendrolimi* analyzed by RT-qPCR. (**C**) Developmental expression profile of the *white* gene in *T. ostriniae* analyzed by RT-qPCR. (**D**) Developmental expression profile of the *laccase 2* gene in *T. ostriniae* analyzed by RT-qPCR. (**E**) Reference developmental stages and native phenotypes of *T. dendrolimi* from the 5th to 12th day post-parasitism (from prepupa to adult). (**F**) Reference developmental stages and native phenotypes of *T. ostriniae* from the 4th to 10th day post-parasitism (from prepupa to adult). The lowercase letters represent the significant difference of the relative gene expression at different developmental stages (*p* < 0.05, Duncan).

To determine the optimal gene silencing period, the temporal expression levels of *white* and *laccase 2* genes across developmental stages in both *Trichogramma* species were analyzed by RT-qPCR. Both genes exhibited constitutive expression throughout ontogeny but displayed distinct stage-specific amplification patterns (Figure 1A–D). Notably, the expression of *white* gene peaked during the early pupal stage (8th day post-parasitism in *T. dendrolimi*) and the prepupal stage (5th day post-parasitism in *T. ostriniae*), necessitating silencing initiation at the prepupal phase to preempt pigment deposition (Figure 1A,C). In contrast, *laccase 2* expression surged during mid-to-late pupal development (9th day post-parasitism in *T. dendrolimi*; 7th day post-parasitism in *T. ostriniae*), requiring dsRNA administration at the early pupal stage to disrupt cuticle sclerotization (Figure 1B,D). Despite host-dependent variations in developmental timelines, these expression phases remained conserved between species.

Anatomical imaging validated these transcriptional patterns: for the *white* gene, images revealed pigmentation accumulation of eye pigments during the prepupal stage, presenting red-colored eyes (Figure 1E); for the *laccase 2* gene, the thickened cuticle and darkening of body coloration occurred during the pupal stage, aligning closely with RT-qPCR results (Figure 1F). This phenotypic-genotypic concordance confirmed that prepupal *white* targeting and early pupal *laccase 2* silencing align with their respective expression maxima, ensuring maximal interference efficacy.

### 3.2. Effects of Different dsRNA Delivery Methods on Survival Rates

**Figure 2:**
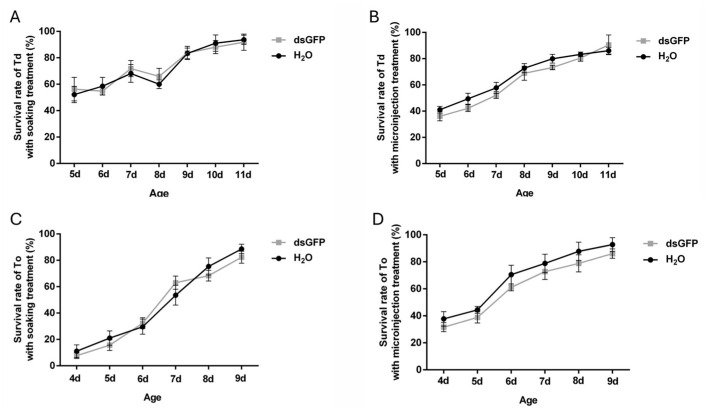
Effects of soaking and microinjection treatments on the survival rate in *T. dendrolimi* (Td) and *T. ostriniae* (To). (**A**) Survival rate of *T. dendrolimi* (from the 5th to 11th day post-parasitism) treated via soaking. (**B**) Survival rate of *T. dendrolimi* (from the 5th to 11th day post-parasitism) treated via microinjection. (**C**) Survival rate of *T. ostriniae* (from the 4th to 9th day post-parasitism) treated via soaking. (**D**) Survival rate of *T. ostriniae* (from the 4th to 9th day post-parasitism) treated via microinjection.

To evaluate the viability of dsRNA delivery methods, survival rates of *T. dendrolimi* and *T. ostriniae* were analyzed following dsRNA administration via soaking or microinjection. Treatments with ddH_2_O and ds*GFP* revealed no significant differences in survival (Figure 2), confirming that dsRNA itself exerted no adverse effects on wasp viability. As development progressed, survival rates for both methods improved across species. For *T. dendrolimi*, soaking demonstrated optimal viability, achieving >50% survival in prepupal and early pupal stage (5th–8th day post-parasitism) and >80% survival in the mid-late pupal stage (9th–11th day post-parasitism), whereas microinjection caused elevated mortality (>60%) at the prepupal stage (Figure 2A,B). Conversely, *T. ostriniae* exhibited species-specific adaptability: prepupae aged 4–5 days post-parasitism tolerated microinjection with survival rates ranging between 30 and 40%, while soaking remained lethal to prepupae with survival rates ranging between 5 and 15%. In pupae aged 6–7 days post-parasitism, microinjection achieved 60–80% survival compared to 30–50% for soaking (Figure 2C,D). These trends necessitated method-stage alignment: soaking was prioritized for *T. dendrolimi* prepupae and pupae, while microinjection was exclusively viable for *T. ostriniae* during the same developmental stages.

### 3.3. Screening for the Optimal Timing of dsRNA Soaking

To establish the optimal soaking duration for dsRNA-mediated RNAi in *Trichogramma* species, *T. dendrolimi* and *T. ostriniae* were treated with dsRNA targeting the *white* and *laccase 2* genes at concentrations of 500 ng/μL and 2000 ng/μL. Silencing efficacy was evaluated over three durations (12 h, 24 h, and 48 h), revealing distinct species- and gene-specific responses.

**Figure 3:**
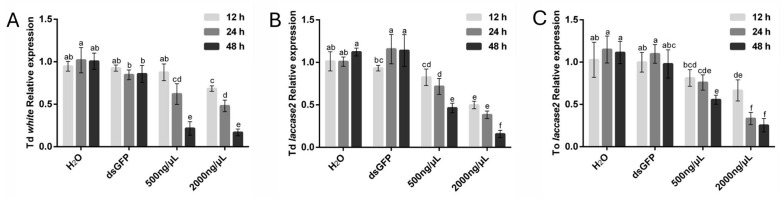
RNAi-mediated knockdown of *white* and *laccase* 2 genes in *T. dendrolimi* (Td) and *T. ostriniae* (To) under varying dsRNA soaking durations. (**A**) The expression of the *white* gene in *T. dendrolimi* after soaking with 500 ng/μL or 2000 ng/μL ds*white*, compared to H_2_O and ds*GFP* controls. (**B**) The expression of the *laccase* 2 gene in *T. dendrolimi* after soaking with 500 ng/μL or 2000 ng/μL ds*laccase 2*, compared to H_2_O and ds*GFP* controls. (**C**) The expression of the *laccase* 2 gene in *T. ostriniae* after soaking with 500 ng/μL or 2000 ng/μL ds*laccase 2*, compared to H_2_O and ds*GFP* controls. The lowercase letters represent the significant difference of the relative gene expression after different treatments (*p* < 0.05, Duncan).

In *T. dendrolimi*, *white* gene silencing exhibited a pronounced time-dependent efficacy (Figure 3A). At 12 h, significant transcript reduction (31.62%) was observed exclusively with the 2000 ng/μL treatment. Extending the soaking period to 24 h enhanced silencing efficiency, achieving reductions of 38.01% (500 ng/μL) and 51.96% (2000 ng/μL). Maximum efficacy was attained at 48 h, with transcript levels reduced by 78.49% (500 ng/μL) and 83.23% (2000 ng/μL), establishing this duration as optimal. In contrast, *T. ostriniae* prepupae exposed to *white* gene-targeting dsRNA displayed an exceptionally high mortality across all tested conditions, precluding reliable data collection. Consequently, *white* gene silencing results for this species were excluded from analysis.

For the *laccase 2* gene, both species exhibited time-dependent silencing efficacy. In *T. dendrolimi*, significant transcript reduction was observed at 12 h with the 2000 ng/μL treatment (50.15%), increasing to 61.66% at 24 h and peaking at 84.29% after 48 h. The 500 ng/μL treatment showed progressive efficacy, achieving reductions of 28.34% at 24 h and 53.69% at 48 h (Figure 3B). Similarly, in *T. ostriniae*, the 2000 ng/μL treatment resulted in 33.47% reduction at 12 h, escalating to 66.72% at 24 h and 74.63% at 48 h. The lower concentration (500 ng/μL) also demonstrated time-dependent improvements, with reductions of 24.06% at 24 h and 44.39% at 48 h (Figure 3C). Notably, 48 h soaking consistently achieved the highest silencing efficiency for both species and concentrations, confirming its status as the optimal duration.

### 3.4. Effects of dsRNA Concentration on White Gene Silencing in Trichogramma Species

**Figure 4:**
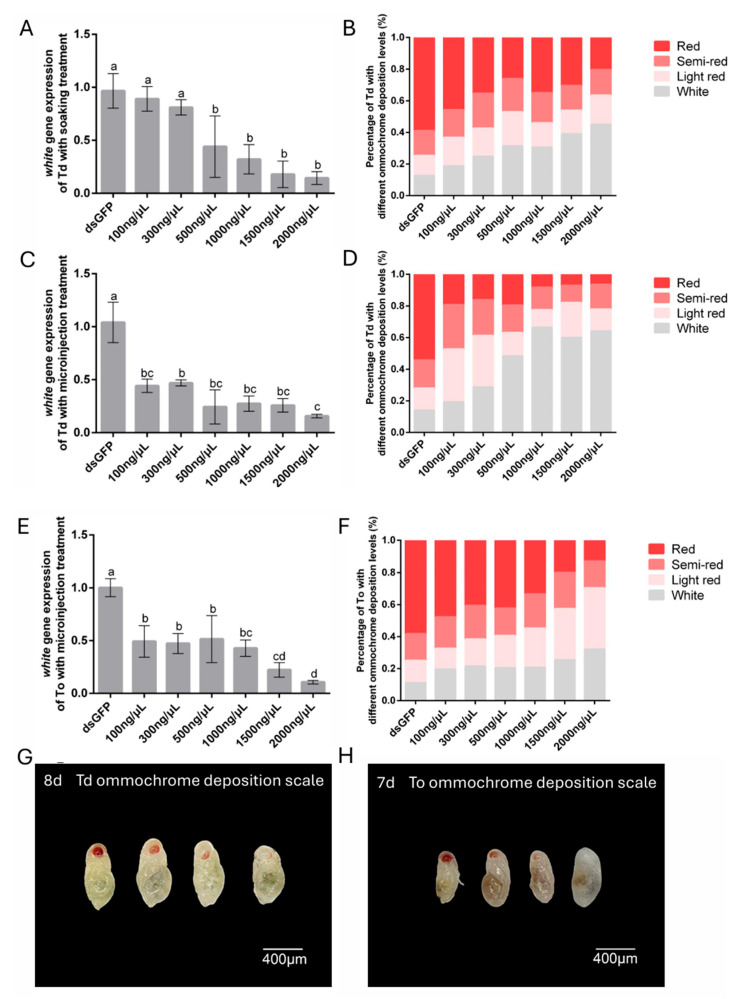
Gene expression and phenotypic changes following RNAi-mediated knockdown of *white* in *T. dendrolimi* (Td) and *T. ostriniae* (To) via soaking and microinjection. (**A**) Relative expression of the *white* gene in *T. dendrolimi* pupae (8th day post-parasitism) following soaking with varying ds*white* concentrations. (**B**) Proportion of eye color phenotypes in *T. dendrolimi* pupae (8th day post-parasitism) after *white* knockdown via soaking. (**C**) Relative expression of the *white* gene in *T. dendrolimi* pupae (8th day post-parasitism) following microinjection with varying ds*white* concentrations. (**D**) Proportion of eye color phenotypes in *T. dendrolimi* pupae (8th day post-parasitism) after *white* knockdown via microinjection. (**E**) Relative expression of the *white* gene in *T. ostriniae* pupae (7th day post-parasitism) following microinjection with varying ds*white* concentrations. (**F**) Proportion of eye color phenotypes in *T. ostriniae* pupae (7th day post-parasitism) after *white* knockdown via microinjection. Phenotype classification in *T. dendrolimi* ((**G**), 8th day post-parasitism) and *T. ostriniae* ((**H**), 7th day post-parasitism): four-tier ommochrome deposition scale: Class I (fully pigmented, red), Class II (partial pigmentation, semi-red), Class III (low pigmentation, light red), and Class IV (unpigmented, white). The lowercase letters represent the significant difference of the relative gene expression after different treatments (*p* < 0.05, Duncan).

To determine the optimal dsRNA concentration for RNAi-mediated *white* gene silencing, *T. dendrolimi* and *T. ostriniae* were treated with six dsRNA concentrations (100–2000 ng/μL) via soaking or microinjection. For the soaking method, significant *white* gene silencing was observed in *T. dendrolimi*, with RT-qPCR analysis revealing a dose-dependent response: 500 ng/μL reduced transcript levels by 55.91%, while 2000 ng/μL induced an 85.61% reduction in transcript levels (Figure 4A). However, soaking proved unsuitable for *T. ostriniae* prepupae (4th day post-parasitism) due to excessive mortality (>90%) (Figure 2C), necessitating microinjection for *white* gene silencing in this species. In contrast, microinjection enabled effective *white* silencing even at lower concentrations. For *T. dendrolimi*, the injection of 100 ng/μL dsRNA induced 55.76% transcript reduction, peaking at 84.29% with 2000 ng/μL (Figure 4C). Similarly, *T. ostriniae* exhibited 50.73% silencing efficiency at 100 ng/μL, rising to 89.36% at 2000 ng/μL (Figure 4E). Notably, both species displayed progressive silencing enhancement with increasing dsRNA concentrations (300–2000 ng/μL), though *T. ostriniae* required higher doses to match *T. dendrolimi*’s efficiency.

Knockdown of the *white* gene caused a significant reduction in eye pigments in *Trichogramma* species (Figures S7 and S8). Further phenotypic analysis revealed a chromatic gradient correlating with silencing efficiency (Figure 4G,H). Untreated controls displayed 12.65% white-eyed pupae after 48-h ds*GFP* soaking, whereas 2000 ng/μL ds*white* treatment via soaking and microinjection increased this proportion to 45.07% and 64.06%, respectively (Figure 4B,D). Notably, microinjection in *T. dendrolimi* outperformed soaking, with 100–300 ng/μL inducing a marked rise in light-red-eyed and white-eyed individuals and ≥500 ng/μL yielding predominantly white-eyed phenotypes (Figure 4B,D). Conversely, *T. ostriniae* exhibited lower phenotypic efficacy, with only 19.63–32.09% white-eyed individuals across 100–2000 ng/μL ds*white* treatments (Figure 4F).

### 3.5. Effects of dsRNA Concentration on Laccase 2 Gene Silencing in Trichogramma Species

**Figure 5:**
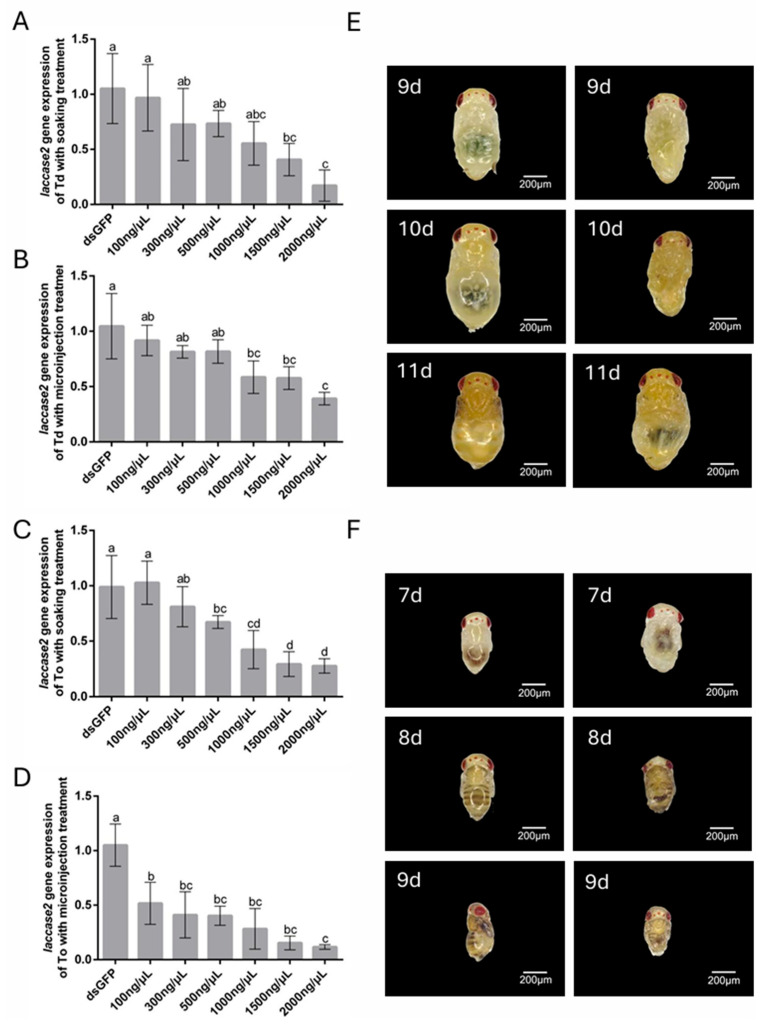
RNAi-mediated knockdown of *laccase 2* in *T. dendrolimi* (Td) and *T. ostriniae* (To) via soaking and microinjection. (**A**) Relative expression of the *laccase 2* gene in *T. dendrolimi* pupa (9th day post-parasitism) following soaking with varying dsRNA concentrations. (**B**) Relative expression of *laccase 2* in *T. dendrolimi* pupa (9th day post-parasitism) following microinjection with varying dsRNA concentrations. (**C**) Relative expression of *laccase 2* in *T. ostriniae* pupa (7th day post-parasitism) following soaking with varying dsRNA concentrations. (**D**) Relative expression of *laccase 2* in *T. ostriniae* pupa (7th day post-parasitism) following microinjection with varying dsRNA concentrations. (**E**) Phenotypic comparison of *T. dendrolimi* pupae (9th–11th day post-parasitism) treated with ds*GFP* (left) and ds*laccase 2* (right). (**F**) Phenotypic comparison of *T. ostriniae* pupae (7th–9th day post-parasitism) treated with ds*GFP* (left) and ds*laccase 2* (right). The lowercase letters represent the significant difference of the relative gene expression after different treatments (*p* < 0.05, Duncan).

The RNAi-mediated targeting of *laccase 2*, a critical regulator of cuticle sclerotization during pupal development, revealed distinct concentration–response dynamics in *T. dendrolimi* and *T. ostriniae*. Unlike pigment-related *white* gene silencing, *laccase 2* knockdown directly compromised structural integrity, with phenotypic severity escalating at higher dsRNA concentrations. Soaking protocols demonstrated robust efficacy across species: *T. dendrolimi* exhibited 88.35% transcript reduction at 2000 ng/μL (Figure 5A), while *T. ostriniae* achieved 73.31% silencing (Figure 5C), both correlating with incomplete cuticle tanning and emergence failure. Microinjection, however, highlighted interspecific divergence—low-dose treatment (100 ng/μL) yielded minimal silencing in *T. dendrolimi* (8.33%, Figure 5B) but moderate efficacy in *T. ostriniae* (49.27%, Figure 5D). At 2000 ng/μL, both methods yielded significant silencing efficiency, with 60.99% and 89.36% transcript reduction in *T. dendrolimi* and *T. ostriniae*, respectively (Figure 5B,D). Phenotypic analysis revealed that *laccase 2* knockdown disrupted cuticle tanning, a critical process initiated at the 7th day post-parasitism. The ds*GFP*-treated controls exhibited normal sclerotization, while *laccase 2*-silenced individuals displayed soft, malformed cuticles 48 h post-treatment in both *Trichogramma* species (Figure 5E,F).

## 4. Discussion

The establishment of RNAi systems in *Trichogramma* wasps has long been constrained by their minute size and host-rearing dependencies. While prior studies focused exclusively on *T. dendrolimi*, our work bridges a critical gap by successfully implementing RNAi in *T. ostriniae*, a superior biocontrol agent used to control *O. furnacalis* traditionally hindered by its reliance on *C. cephalonica* eggs and sub-0.5 mm body size. By tailoring delivery methods to species-specific physiological constraints, we demonstrate that soaking remained effective for pupal-stage gene silencing in both *T. dendrolimi* and *T. ostriniae*, whereas microinjection enables robust gene silencing in *T. ostriniae* prepupae—a breakthrough that expands functional genomics tools to non-model *Trichogramma* species. This advancement not only resolves the historical neglect of *T. ostriniae* in molecular studies but also provides a framework for adapting RNAi protocols to microhymenopteran parasitoids with diverse host preferences.

The selection of dsRNA delivery methods in *Trichogramma* wasps hinges on balancing technical feasibility, species-specific physiological constraints, and silencing efficiency. Microinjection, the predominant RNAi method in parasitoid wasps [20,21], offers precise tissue-targeted delivery with controlled dsRNA concentration but requires specialized equipment (e.g., microprocessor-controlled injectors) and incurs operational complexity compared to non-invasive methods. While studies report dsRNA injection amounts ranging widely from 500 ng/μL to 6 μg/μL in other parasitoids [22,23], our results demonstrate that *Trichogramma* species achieve significant gene silencing at lower concentrations. For instance, the microinjection of 100 ng/μL dsRNA induced 50.8% transcript reduction in the *laccase 2* gene in *T. ostriniae* and 57.46% and 49% transcript reduction in the *white* gene in *T. dendrolimi* and *T. ostriniae*, respectively, highlighting exceptional dsRNA bioavailability in these microhymenopterans. Intriguingly, increasing dsRNA concentrations to 200–1500 ng/μL were not effective in enhancing silencing efficacy, consistent with the hypothesis of a saturation threshold in the RNAi machinery [10], which underscores the efficiency of low-dose microinjection in *Trichogramma*.

In contrast, the non-invasive soaking method offers operational simplicity by facilitating passive dsRNA uptake through cuticular or spiracular pathways during the pupal stage [24,25], eliminating the mechanical trauma associated with microinjection. However, its efficacy is strongly species-dependent due to variations in osmotic tolerance. For *T. dendrolimi*, which is industrially reared in *A. pernyi* eggs, soaking at 2000 ng/μL achieved >80% survival and silencing efficiency for both *white* and *laccase 2* genes, aligning well with large-scale experimental requirements. Conversely, the smaller-bodied *T. ostriniae* (0.3 mm) exhibited severe sensitivity to this passive absorption mechanism, with prepupal survival dropping below 20% under identical conditions, likely due to osmotic stress. Notably, at the elevated concentration of 2000 ng/μL, soaking matched microinjection in silencing efficacy across both species, demonstrating its potential as a viable alternative where species-specific physiological barriers are absent.

Temporal dynamics further modulate RNAi efficacy during soaking, necessitating a balance between silencing optimization and developmental viability. In *T. dendrolimi*, soaking with 2000 ng/μL ds*white* for 24 h reduced *white* and *laccase 2* transcripts by 51.95% and 61.66%, respectively. Extending the soaking period to 48 h further enhanced *white* silencing to 83.23%, though *laccase 2* efficiency slightly declined to 84.29%, accompanied by an increase in mortality. This divergence suggests gene-specific susceptibility to prolonged dsRNA exposure, potentially linked to differential expression kinetics or pathway saturation. Similar trends were observed in *Microplitis mediator*, where *MmedOR49* silencing efficiency rose from 30% (24 h) to 50% (48 h) under equivalent conditions [20], underscoring the universality of time-dependent RNAi efficacy in parasitoids. The progressive improvement in silencing with extended soaking likely stems from cumulative dsRNA absorption through cuticular or spiracular pathways, enabling the sustained saturation of RNAi machinery components. This contrasts starkly with microinjection, where a single bolus dose degrades or dilutes over time, requiring precise alignment with transcriptional peaks [3].

RNAi efficiency also exhibits substantial variability among genes within the same insect species, even when uniform delivery methods are employed [11]. For instance, in *Aphidius ervi*, dsRNA targeting *AeSPH1* induced a significant reduction in its transcript levels, whereas dsRNA targeting *AeSPN1* failed to alter *AeSPN1* expression under identical experimental conditions [26]. Similarly, in *T. dendrolimi*, the silencing efficacy of the *white* gene consistently surpassed that of the *laccase 2* gene across both soaking and injection delivery methods. These observations underscore the intrinsic gene-specific sensitivity to RNAi, which may arise from factors such as transcript abundance, dsRNA accessibility, or sequence-specific secondary structures [27,28]. This phenomenon is not unique to hymenopterans. In coleopterans, broad-scale screens of dsRNAs targeting 290 genes in *Diabrotica virgifera virgifera* revealed marked differences in efficacy, with the LC_50_ values of the 17 most effective dsRNAs varying by nearly 100-fold [29]. Similarly, in the Colorado potato beetle (*Leptinotarsa decemlineata*), transcript suppression levels ranged from approximately 60% to 93% depending on the target gene [30]. Such variability emphasizes the necessity of empirical gene screening and mechanistic studies to optimize RNAi applications [3,11,31].

The successful implementation of RNAi in both *T. dendrolimi* and *T. ostriniae* demonstrates that the optimized framework in this study holds significant potential for broader application across the *Trichogramma* genus. Crucially, the observed interspecies divergence between these species underscores that protocol transferability fundamentally depends on accommodating species-specific physiological constraints. The comparative analysis reveals that optimal dsRNA delivery method selection is dictated by developmental tolerance, where robust species like *T. dendrolimi* achieve efficient silencing via non-invasive soaking during pupal stages, while *T. ostriniae* prepupae necessitate microinjection to maintain viability. Beyond delivery routes, key operational parameters require species-tailored optimization; although 2000 ng/µL dsRNA proved effective here, optimal concentrations for silencing efficiency and minimal toxicity may vary across species targets or developmental stages and must be empirically determined through dose–response testing. Similarly, treatment parameters including soaking duration and microinjection volume demand calibration based on biological factors like body size and cuticle permeability. This dual emphasis on physiological adaptation and parametric calibration establishes a replicable blueprint for extending RNAi methodologies to previously intractable *Trichogramma* species.

Our study advances RNAi methodology in *Trichogramma* wasps by overcoming species-specific limitations and elucidating the intricate interplay between delivery methods, the temporal dynamics of gene silencing, and gene-specific susceptibility to RNAi. Specifically, by systematically addressing the technical bottlenecks in dsRNA delivery that have long hindered functional genomics research in minute-bodied parasitoids, this work establishes a robust framework for precise genetic manipulation. These advancements not only establish an efficient RNAi system tailored to *Trichogramma* biology but also directly enable targeted genetic interventions to enhance traits critical for biocontrol efficacy, providing a crucial technical support for pest control and management.

## 5. Conclusions

In this study, through different dsRNA delivery methods and phenotype analysis of two genes in *Trichogramma* wasps, we found that both methods are applicable for *T. dendrolimi*, with a 48 h soaking treatment achieving approximately 85% gene silencing efficiency. For *T. ostriniae,* with a smaller body size during the prepupal stage, the microinjection method was more suitable, while the soaking method was more convenient during the pupal stage. The microinjection method showed a significant silencing efficiency at a dsRNA concentration of 500 ng/μL, with the strongest silencing efficiency observed at a concentration of 2000 ng/μL. When using 1000–2000 ng/μL dsRNA for the soaking methods, it could exhibit the same silencing effect as microinjection methods. This optimized system transcends the prior limitation of RNAi applications confined to *T. dendrolimi* within *Trichogramma* species, establishing a robust platform for systematically elucidating molecular mechanisms governing parasitic behaviors in these wasps.

## Figures and Tables

**Table 1:** Primers used in RT-qPCR of *white* and *laccase 2* genes.

Primer Name	Primer Sequences
*white*-F	TTGACACCATTGCGTGTACT
*white*-R	CATCCATCCAAAAGTGTTCC
*laccase 2*-F	CTGCTTCACCGAGAGTTCAA
*laccase 2*-R	AGGAAGTGGCAATGGAAGAG
*GAPDH*-F	AGATCAAGGCCAAGGTCAAG
*GAPDH*-R	AGTGGTTGTCACCGATGAAG

**Table 2:** Primers used for dsRNA synthesis of *white* and *laccase 2* genes.

Primer Name	Primer Sequences
*white*-DF	TAATACGACTCACTATAGGGGCCCTTGTTCGAATGGATAA
*white*-DR	TAATACGACTCACTATAGGGTTGACAATCGAAATCCACGA
*laccase 2*-DF	TAATACGACTCACTATAGGGACGATCTCACCGTCATAGCC
*laccase 2*-DR	TAATACGACTCACTATAGGGGAACTGCTCGGGTGGTATGT

## Data Availability

The original contributions presented in this study are included in the article/Supplementary Material. Further inquiries can be directed to the corresponding authors.

## Supplementary Materials

The following supporting information can be downloaded at: https://www.mdpi.com/article/10.3390/insects16070673/s1, Figure S1. Sequence alignment analysis of *white* genes from *T. dendrolimi* and other species; Figure S2. Sequence alignment analysis of *laccase 2* genes from *T. dendrolimi* and other species; Figure S3. A detailed dissection procedure of *T. dendrolimi* pupae from *Antheraea pernyi* eggs; Figure S4. A detailed dissection procedure of *T. ostriniae* pupae from *Ostrinia furnacalis* eggs. Figure S5. Soaking-mediated RNAi in *Trichogramma* wasps. Figure S6. Microinjection-mediated RNAi in *Trichogramma* wasps; Figure S7. Phenotypes of *T. dendrolimi* before and after *white* gene silencing; Figure S8. Phenotypes of *T. ostriniae* before and after *white* gene silencing. Figure S9: Comparison of *white* gene silencing fragments of *T. dendrolimi* and *T. ostriniae*. Figure S10: Comparison of *laccase 2* gene silencing fragments of *T. dendrolimi* and *T. ostriniae.*
