# Supplementary material for: Development of a Stage- and Species-Specific RNAi System for Molecular Insights in Trichogramma Wasps
Source: Insects. 2025 Jun 27;16(7):673. doi: 10.3390/insects16070673 (PMC12294811; doi:10.3390/insects16070673)
Supplement: Supplementary file 1 [file insects-16-00673-s001.zip › insects-3709587-supplementary.pdf]

for:

Development of a Stage- and Species-Specific RNAi System for Molecular Insights in *Trichogramma* Wasps

Zelong Yang [1], Yan Lu [1], Zhuo Jiang [1], Xilin Jiao [1], Han Lin [1], Wanning Jiang [1], Wenmei Du[1], Xue Zhang [1], Zhao Peng [2], Junjie Zhang [1], Xiao Wang [1*], Ying Hu [1*]

1. Engineering Research Center of Natural Enemies, Institute of Biological Control, Jilin Agricultural University, Changchun, 130118, China. 17731008475@163.com (Z.Y.); luyan20020624@163.com (Y.L.); jzjz8968@163.com (Z.J.); j18943100339@163.com (X.J.); 15568628611@163.com (H.L.); winniejiang1008@163.com (W.J.); 280114191@qq.com (W.D.); zhangxue871013@163.com (X.Z.); zhangjunjie9777@jlau.edu.cn (J.Z.); wangxiao@jlau.edu.cn (W.X.), huying@jlau.edu.cn (Y.H.)

2. Department of Plant Pathology, College of Plant Protection, Jilin Agricultural University, Changchun 130118, China. zpeng21@jlau.edu.cn (P.Z.)

* Corresponding author: Xiao Wang, Ying Hu

E-mail address: wangxiao@jlau.edu.cn, huying@jlau.edu.cn

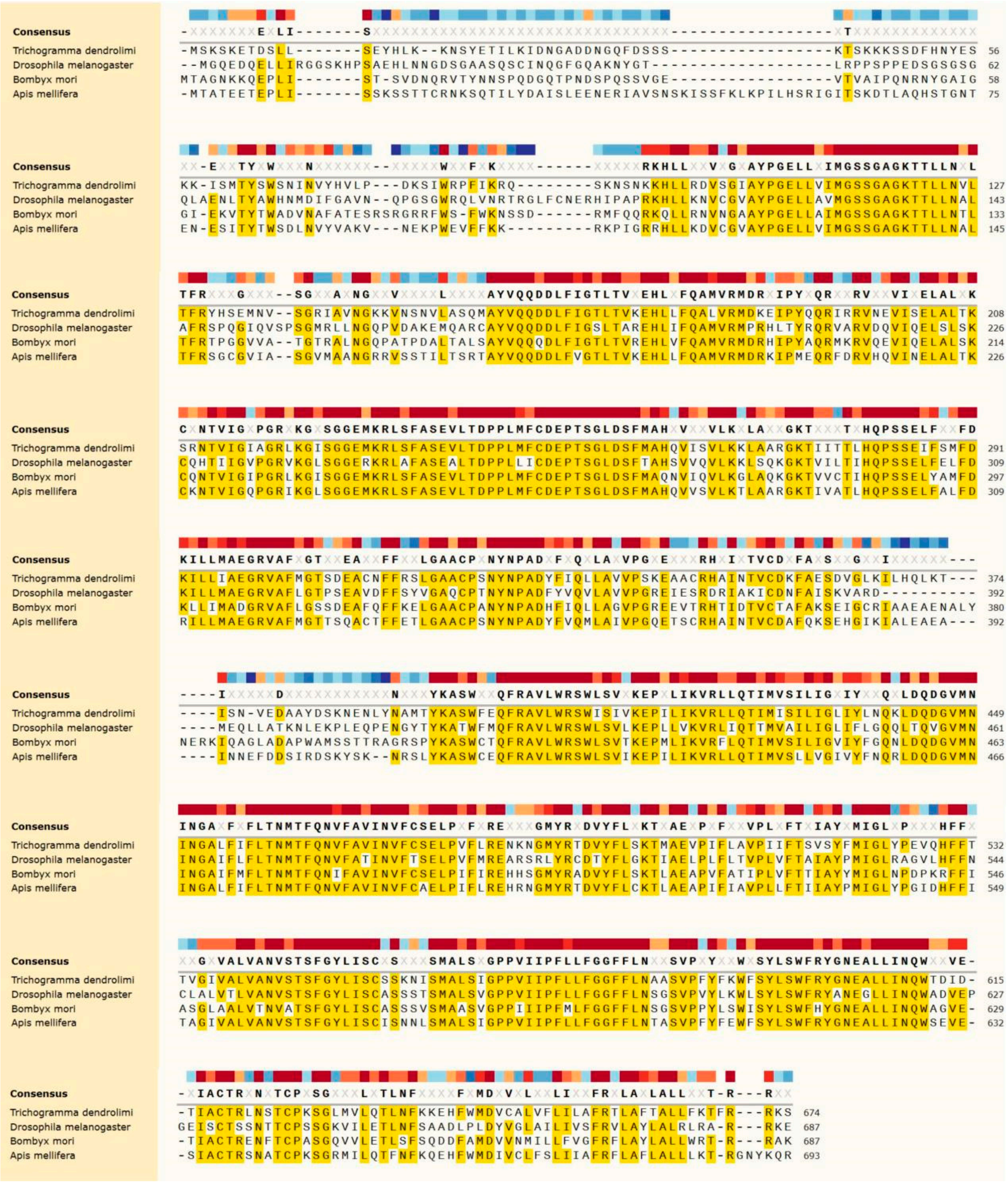

Supplementary Figure S1. Sequence alignment analysis of *white* genes from *T. dendrolimi*, *Drosophila melanogaster*, *Bombyx mori* and *Apis mellifera*.

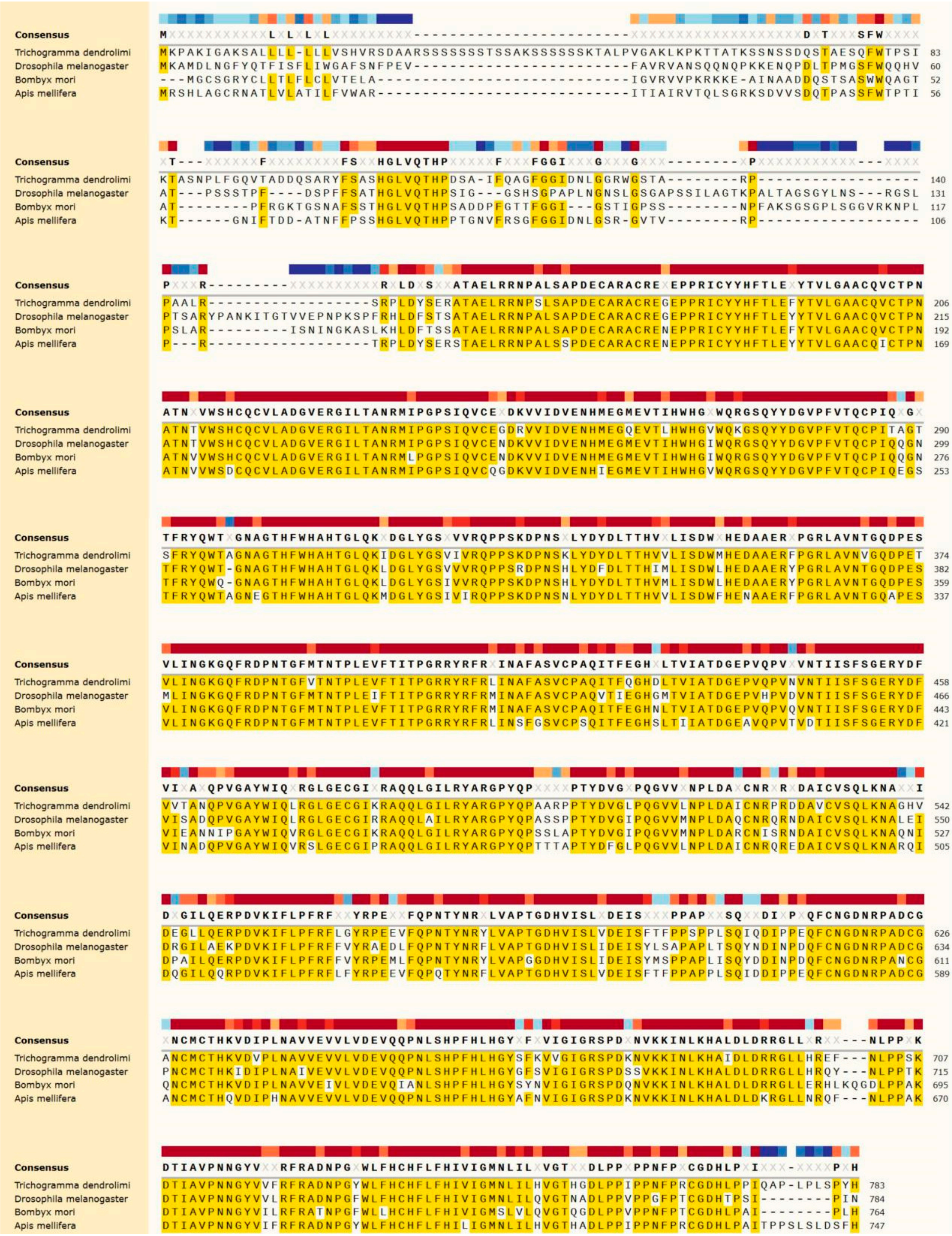

Supplementary Figure S2. Sequence alignment analysis of *laccases 2* genes from *T. dendrolimi*, *Drosophila melanogaster*, *Bombyx mori* and *Apis mellifera*.

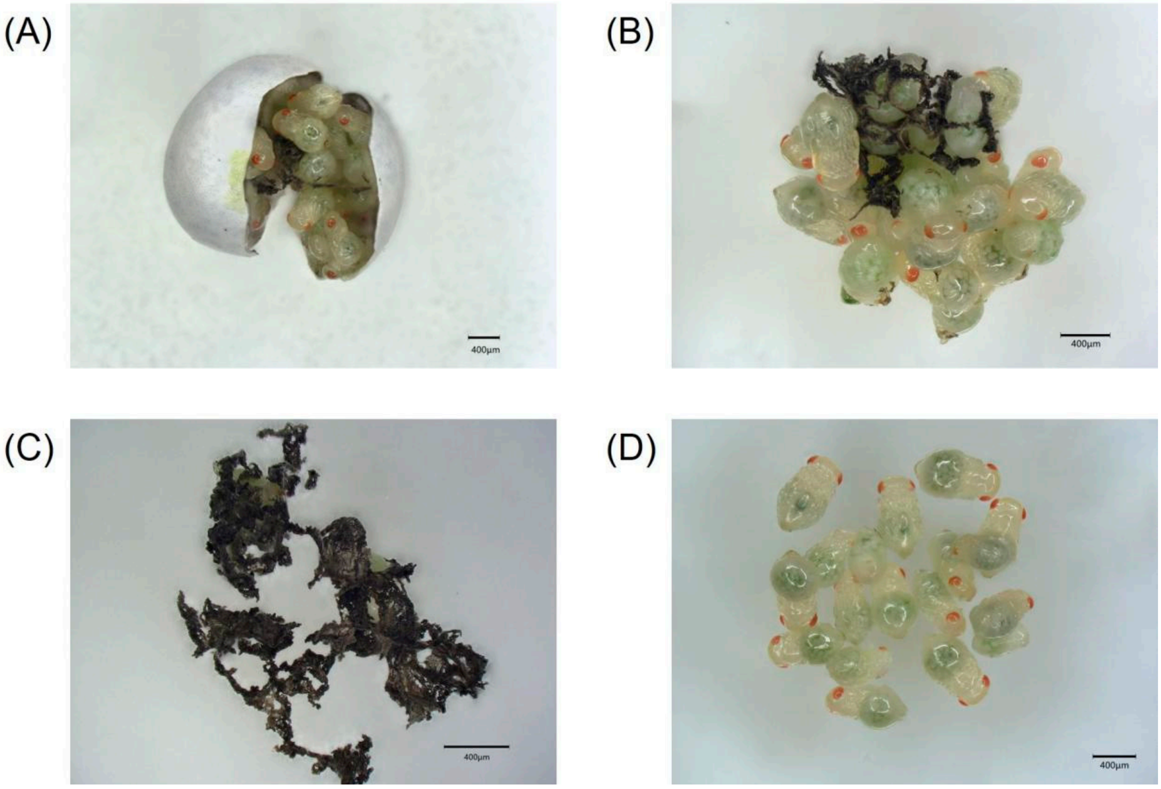

Supplementary Figure S3. Dissection-based isolation of *Trichogramma dendrolimi* Pupae from *Antheraea pernyi* Eggs. (A) Bisected *A. pernyi* egg halves revealing internal *T. dendrolimi* pupa; (B) Dissected *T. dendrolimi* pupa from an *A. pernyi* egg; (C) Removed hydrophobic black matrix encapsulating *T. dendrolimi* pupa in *A. pernyi* egg; (D) *T. dendrolimi* pupa devoid of hydrophobic black matrix post-removal; Scale bar = 400 μm.

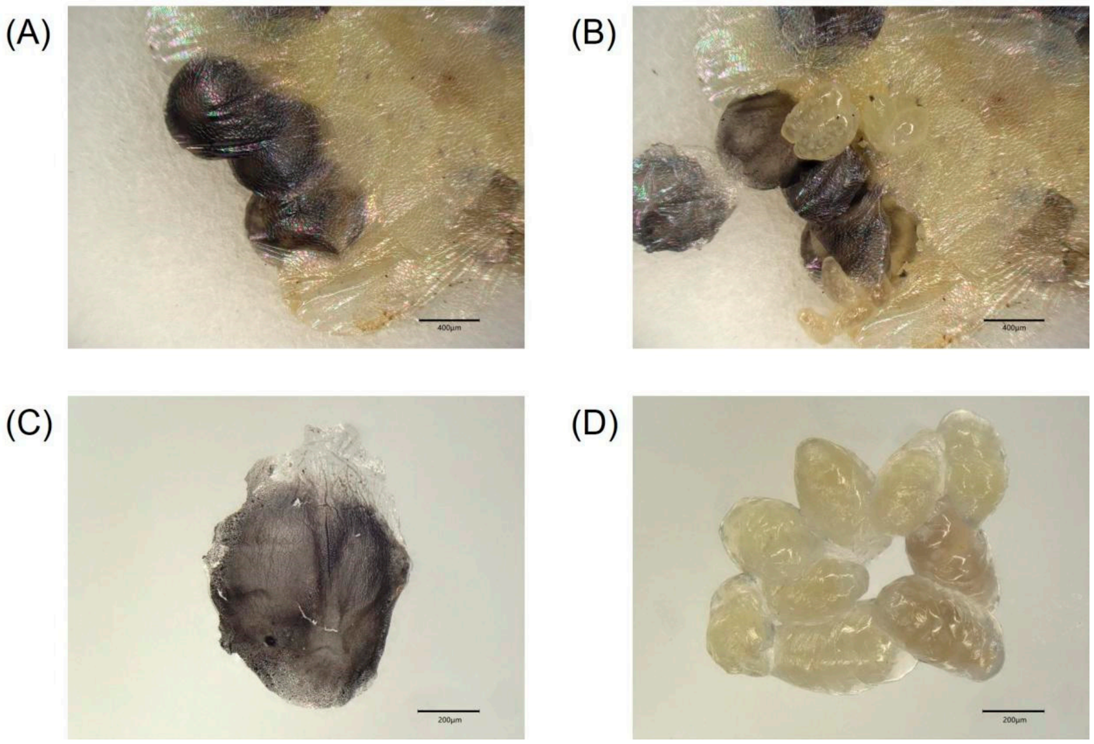

Supplementary Figure S4. Dissection-based isolation of *Trichogramma ostriniae* Pupae from *Ostrinia furnacalis* Eggs. (A) *O. furnacalis* egg parasitized by *T. ostriniae* (black coloration indicates parasitized eggs); Scale bar = 400 μm; (B) Dissection of *T. ostriniae* pupae from *O. furnacalis* eggs; Scale bar = 400 μm; (C) Chorionic remnants of *O. furnacalis* host egg after dissection; Scale bar = 200 μm; (D) Dissected *T. ostriniae* pupa isolated from *O. furnacalis* egg chorion; Scale bar = 200 μm.

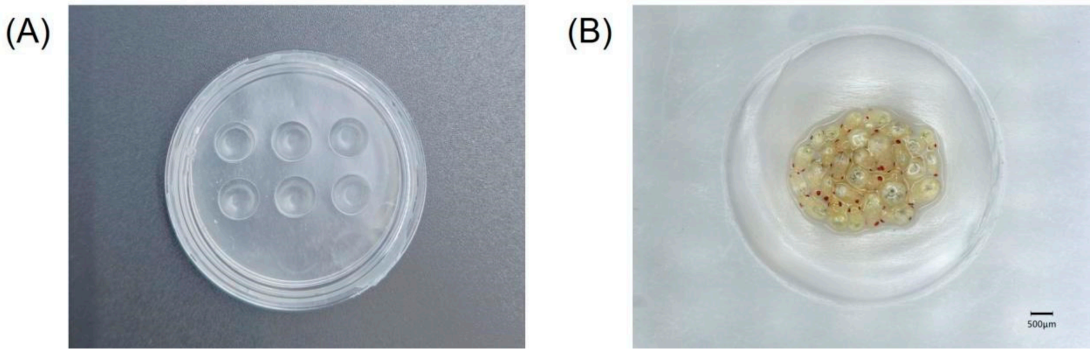

Supplementary Figure S5. Soaking-mediated RNAi in *Trichogramma* wasps. (A) Preparation of artificial host eggs; (B) *Trichogramma* pupae that soaked in dsRNA solutions within artificial host eggs.

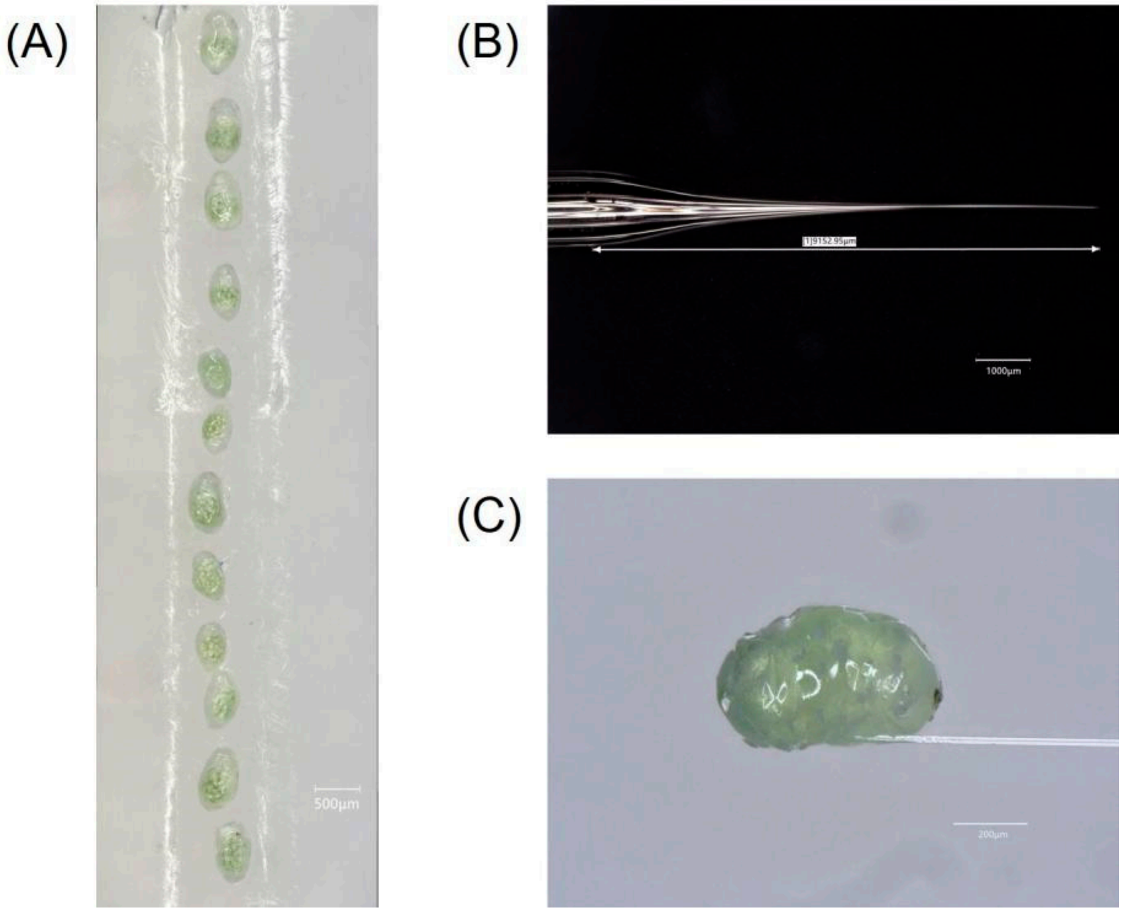

Supplementary Figure S6. Microinjection-mediated RNAi in *Trichogramma* wasps. (A) Mounting *Trichogramma* pupae on an agar substrate for microscopic manipulation; (B) Glass needle used for microinjection; (C) Microinjection into a *Trichogramma* pupa.

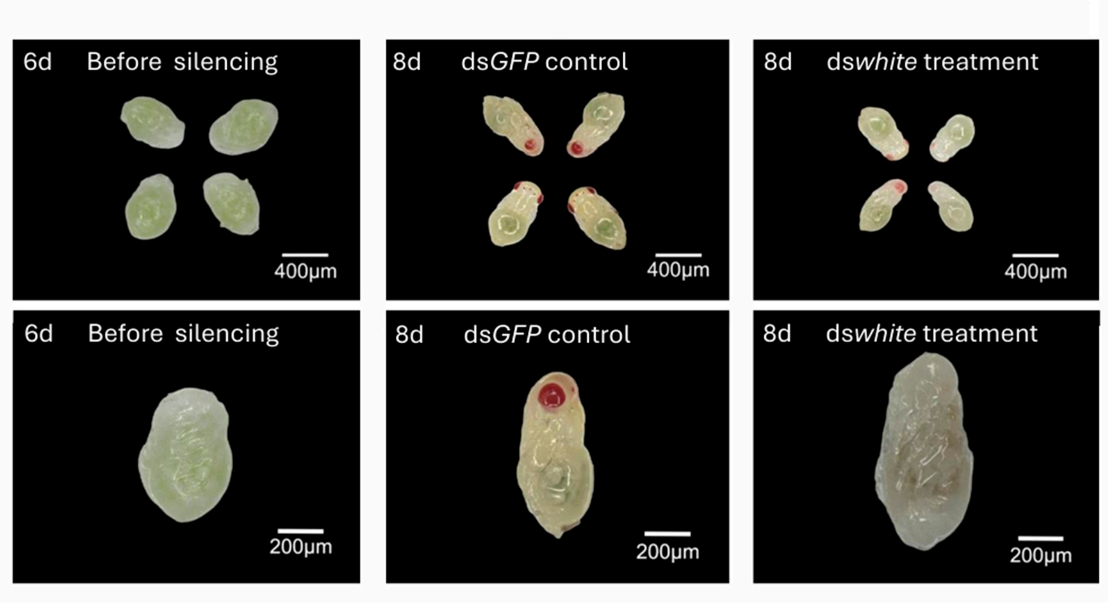

Supplementary Figure S7. Phenotypes of *T. dendrolimi* before and after *white* gene silencing. Left panel: The synchronized prepupa (6th day post-parasitism) before dsRNA treatment. Middle panel: After 48 hours dsRNA treatment, pupa (8th day post-parasitism) treated with ds*GFP*. Right panel: After 48 hours dsRNA treatment, pupa (8th day post-parasitism) treated with ds*white*.

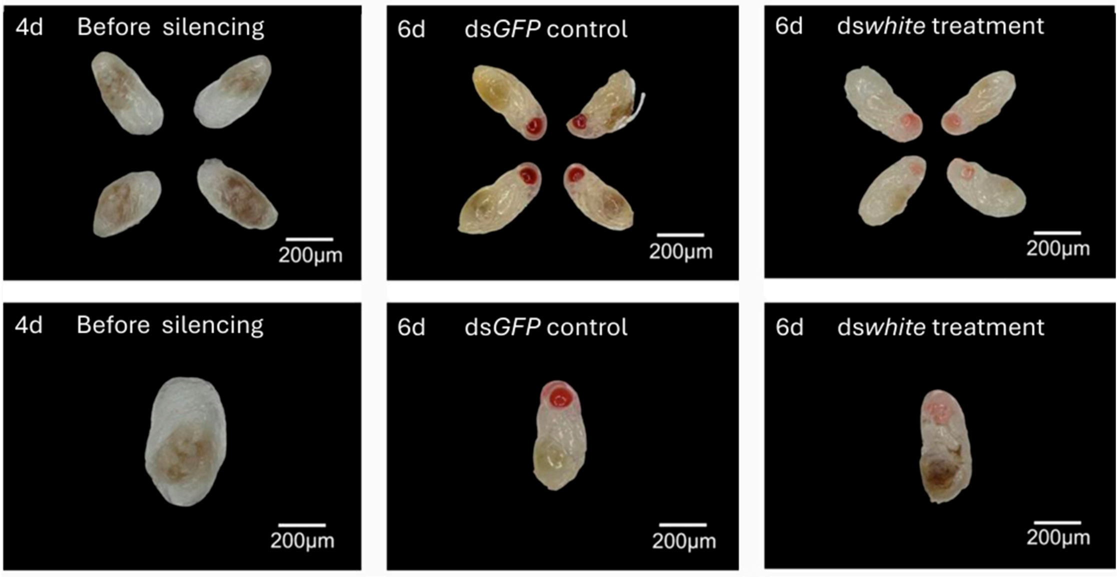

Supplementary Figure S8. Phenotypes of *T. ostriniae* before and after *white* gene silencing. Phenotypes of *T. ostriniae* before and after *white* gene silencing. Left panel: The synchronized prepupa (4th day post-parasitism) before dsRNA treatment. Middle panel: After 48 hours dsRNA treatment, pupa (6th day post-parasitism) treated with ds*GFP*. Right panel: After 48 hours dsRNA treatment, pupa (6th day post-parasitism) treated with ds*white*.

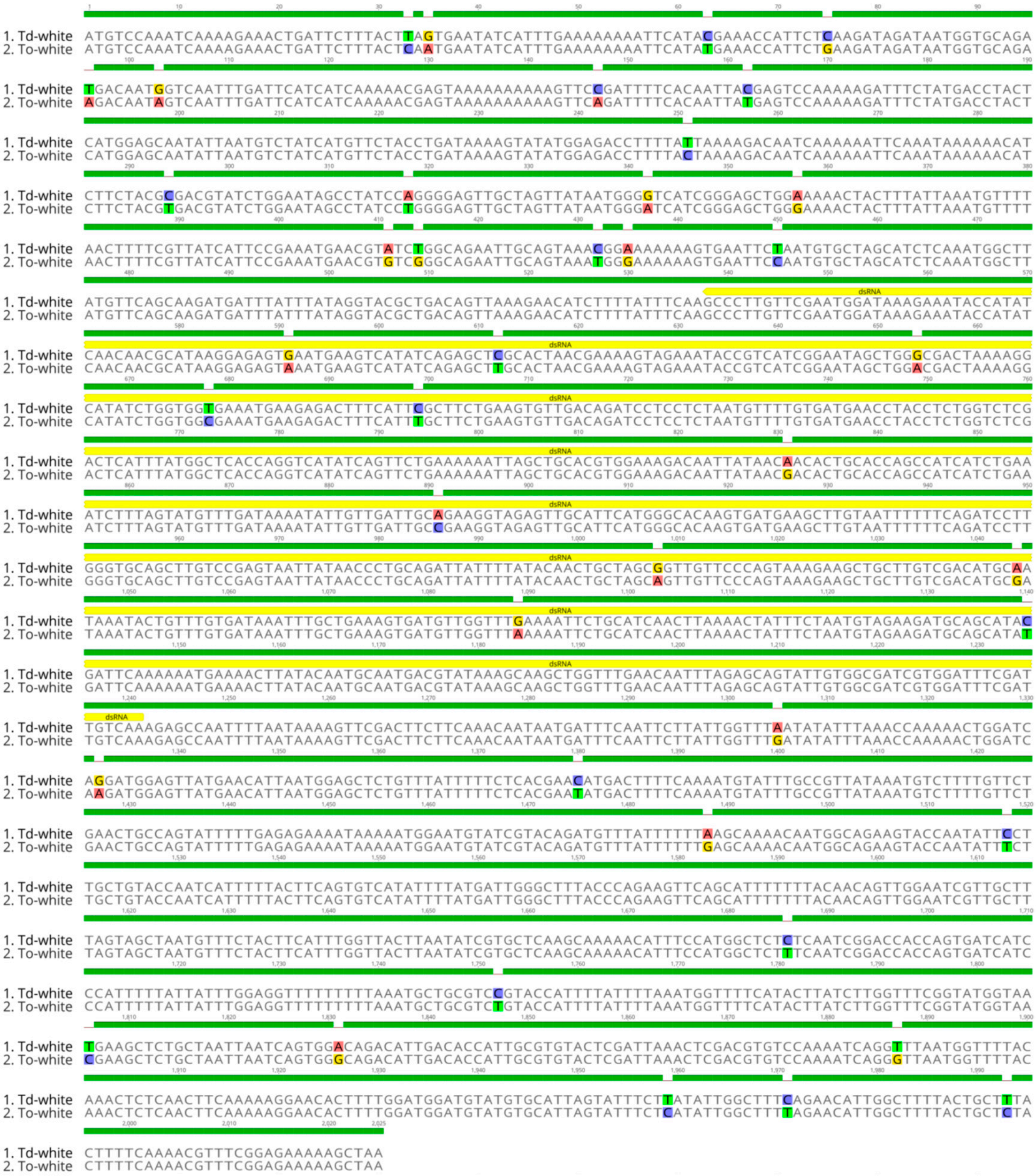

Supplementary Figure S9. Pairwise alignment of the *white* genes of *T. dendrolimi* and *T. ostriniae*. The region corresponding to the designed RNAi fragment is highlighted in yellow.

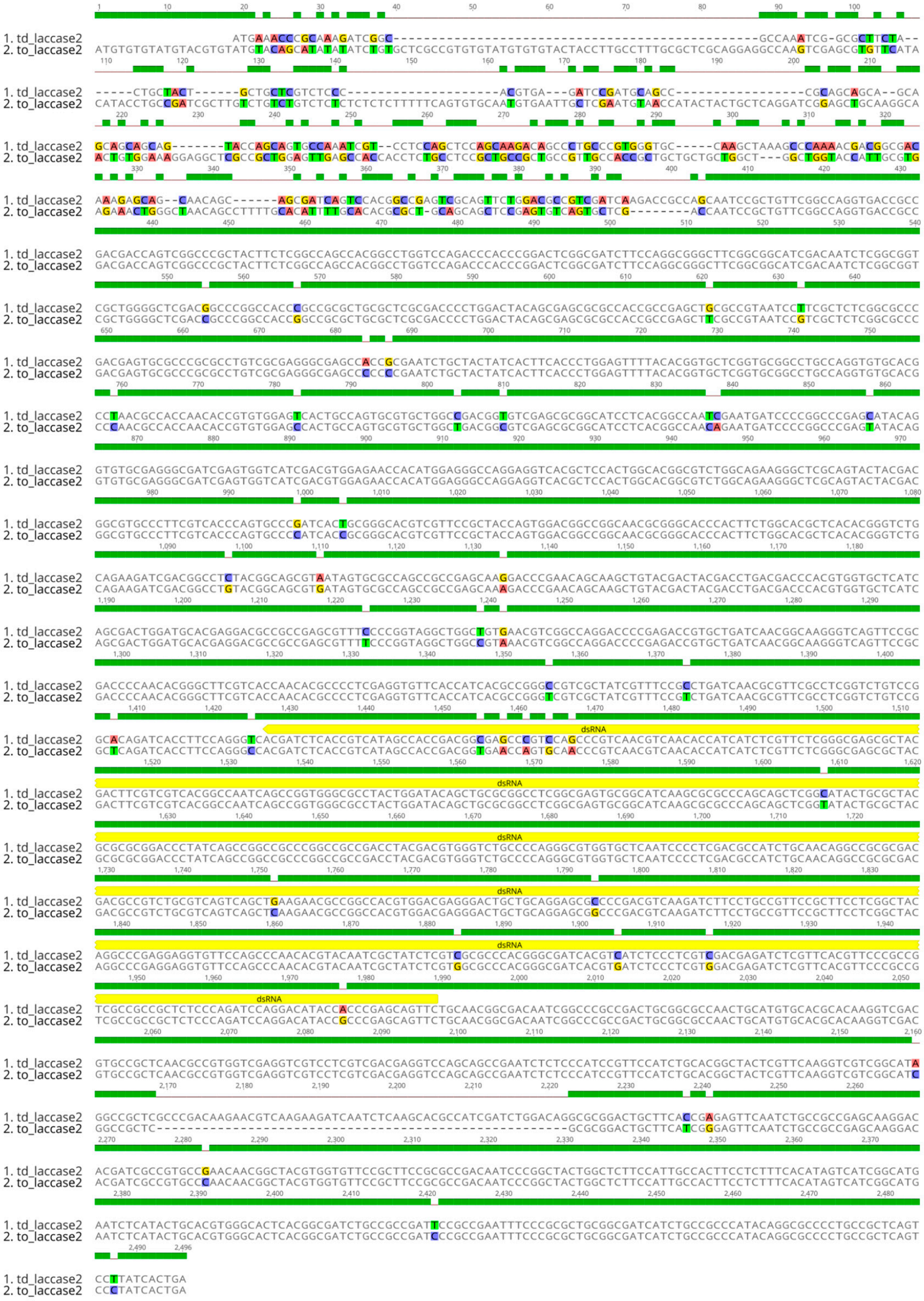

Supplementary Figure S10. Pairwise alignment of the *laccase 2* genes of *T. dendrolimi* and *T. ostriniae*. The region corresponding to the designed RNAi fragment is highlighted in yellow.
